# Human iPS cell model of type 3 long QT syndrome recapitulates drug-based phenotype correction

**DOI:** 10.1007/s00395-016-0530-0

**Published:** 2016-01-23

**Authors:** Daniela Malan, Miao Zhang, Birgit Stallmeyer, Jovanca Müller, Bernd K. Fleischmann, Eric Schulze-Bahr, Philipp Sasse, Boris Greber

**Affiliations:** Institute of Physiology I, Life & Brain Center, University of Bonn, Sigmund-Freud-Str. 25, 53127 Bonn, Germany; Human Stem Cell Pluripotency Group, Max Planck Institute for Molecular Biomedicine, 48149 Münster, Germany; Chemical Genomics Centre of the Max Planck Society, 44227 Dortmund, Germany; Department of Cardiovascular Medicine, Institute for Genetics of Heart Diseases (IfGH), University Hospital Münster, 48149 Münster, Germany

**Keywords:** Human iPS cells, Cardiac disease modeling, Type 3 long-QT syndrome, Drug testing

## Abstract

Long QT syndrome is a potentially life-threatening disease characterized by delayed repolarization of cardiomyocytes, QT interval prolongation in the electrocardiogram, and a high risk for sudden cardiac death caused by ventricular arrhythmia. The genetic type 3 of this syndrome (LQT3) is caused by gain-of-function mutations in the *SCN5A* cardiac sodium channel gene which mediates the fast Na_v_1.5 current during action potential initiation. Here, we report the analysis of LQT3 human induced pluripotent stem cell-derived cardiomyocytes (hiPSC-CMs). These were generated from a patient with a heterozygous p.R1644H mutation in *SCN5A* known to interfere with fast channel inactivation. LQT3 hiPSC-CMs recapitulated pathognomonic electrophysiological features of the disease, such as an accelerated recovery from inactivation of sodium currents as well as action potential prolongation, especially at low stimulation rates. In addition, unlike previously described LQT3 hiPSC models, we observed a high incidence of early after depolarizations (EADs) which is a trigger mechanism for arrhythmia in LQT3. Administration of specific sodium channel inhibitors was found to shorten action and field potential durations specifically in LQT3 hiPSC-CMs and antagonized EADs in a dose-dependent manner. These findings were in full agreement with the pharmacological response profile of the underlying patient and of other patients from the same family. Thus, our data demonstrate the utility of patient-specific LQT3 hiPSCs for assessing pharmacological responses to putative drugs and for improving treatment efficacies.

**Electronic supplementary material:**

The online version of this article (doi:10.1007/s00395-016-0530-0) contains supplementary material, which is available to authorized users.

## Introduction

A diagnostic hallmark of LQTS is a prolonged QT interval in the electrocardiogram (ECG) of patients, resulting from impaired myocellular repolarization during action potential (AP) generation. Approximately 5–10 % of LQTS patients are carriers of a gain-of-function mutation in *SCN5A*, the gene encoding the α-subunit of the cardiac sodium channel (LQT3 subtype). These mutations result in an enhanced recovery from channel inactivation and re-activation during the plateau phase of the action potential [2, 21, 27]. Thus, the persistent inward sodium current counteracts cardiac repolarization resulting in prolonged action potentials as well as in the induction of EADs which are key triggers of ventricular tachycardia [4, 12, 18, 30].

In clinical terms, LQT3 patients often exhibit QT interval prolongation at lower heart rates and, consequently, have an increased risk for cardiac events during rest or sleep [23]. This is in contrast to other LQTS subtypes such as LQT1, where cardiac events typically occur at increased heart rates, that is, during physical or emotional (i.e. adrenergic) stress [5, 24]. Therefore, while β-blocker therapy alone can be sufficient for treating LQT1 patients, effective treatment options in LQT3 patients may require additional measures and hence, the best suited pharmacological treatments are still being explored [20]. Moreover, numerous mutations in *SCN5A* have been identified to cause distinct disease phenotypes [18, 22], and drug efficacy may be mutation-specific, suggesting that treatments need to be tailored to a given specific gene defect [19]. In vitro drug screening systems may therefore aid in predicting therapeutic efficacy. LQT3 models based on patient-specific hiPSCs have been shown to recapitulate key electrophysiological disease features such as increased late Na^+^ currents and prolonged action potentials at the single-cell level [15, 29]. However, more macroscopic phenotypes like induced arrhythmia or spontaneous EADs have not been reported in these or related studies [6, 9].

To re-investigate this latter point and potentially assess pharmacological response profiles, we have established a patient-derived hiPSC model harbouring a heterozygous *SCN5A* mutation (p.R1644H) mutation that is known to cause LQT3 [31]. Disease pathogenesis of this typical LQT3 mutation is due to disperse sodium channel re-openings following fast initial inactivation [2, 7, 30]. The amplitude of the late Na^+^ current is small (<5 %) when compared to that of the initial inward one. Nonetheless, the premature recovery from inactivation of the Na^+^ current will counteract cardiomyocyte repolarization, to macroscopically cause a long QT phenotype [2]. Interestingly, besides displaying corresponding electrophysiological phenotypes, cardiac syncytia of R1644H hiPSC-CMs showed spontaneous EADs which are key triggers of arrhythmia in LQT3 patients [12]. EADs could be abolished by treating mutant hiPSC-CMs with the same drug that was successfully used to treat the underlying patient. Our data hence suggest patient-specific hiPSC-CMs may serve as a predictive system for drug assessment in LQT3 personalized medicine.

## Materials and methods

### Clinical patient history and data generation

In 2006, members of a large family (*n* = 23) with congenital LQTS presented first in our outpatient service. In one family branch, a sudden infant death occurred during the second month of age. Despite lack of pathological investigation or molecular autopsy, a sudden infant death syndrome (SIDS) caused by LQTS seemed likely, since her mother and a sister were also affected by LQTS. Genotyping and family cascade screening of all family members was then initiated. All family members who participated in the study gave written informed consent before genetic and clinical investigations, in accordance with the last version of the Declaration of Helsinki (World Medical Association and R281) and with recommendations by the local ethics committee. Briefly, ECG analysis was performed using conventional 12-lead ECG recordings and standard lead positions (paper speed: 50 mm/s). Heart rate-corrected QT intervals (QTc) were calculated using Bazett’s formula. Genomic DNA was isolated from blood lymphocytes by standard semiautomatic procedures (QIAcube, Qiagen). Locus-specific DNA sequencing was carried out by investigating the major genes relevant for LQTS, *KCNQ1* (LQT1), *KCNH2* (LQT2) and, subsequently, *SCN5A* (LQT3). The patients’ sequence data were compared to the genomic reference (NM_198056.2). Amino acid annotations were based on the corresponding human protein sequences (Locus Reference Genome: LRG_289p1). Alamut annotation software (Interactive Biosoftware) was used for mutation nomenclature.

### Generation and characterization of hiPSCs

Skin punch biopsies were obtained from one affected LQT3 patient of this family as well as from a healthy control individual, following written informed consent and approval by the medical ethics committee of the University of Münster. Fibroblasts that grew out from the dermal tissue were expanded in conventional serum-containing culture media, and subjected to cellular reprogramming following Melton’s protocol [13]. Retroviruses were produced in 293T cells using Fugene 6 transfection with Addgene plasmids 8454 (VSV-G envelope), 8449 (packaging plasmid), 17217 (OCT4), 17218 (SOX2), and 17219 (KLF4) [28]. After retroviral infection of fibroblasts and culture in conventional hESC media with 0.5 mM of valproic acid, emerging colonies were manually picked and expanded. Several cell lines displaying typical hESC morphology and growth characteristics were further characterized according to standard assays [11]. In brief, the heterozygous LQT3 mutation was confirmed using conventional gPCR, cloning and sequencing (Table S2). Transgene silencing was monitored using primers given in Table S2. Karyotypes were assessed based on chromosome counting using standard procedures. The surface marker SSEA4 was detected using standard immunocytochemistry procedures (Millipore #90231, 1:50). hESC marker gene expression was monitored using RT-qPCR analysis as described [11], using M-MLV (Affymetrix #78306) with dT_15_ priming, iTaq™ Universal SYBR Green Supermix (BioRad #172-5853), and RPS16/RPL37A as housekeeping controls (Table S2). Global transcriptome profiling in comparison to hESCs was performed using TotalPrep™ RNA Amplification kits (Life Technologies #AMIL1791) and Illumina human-12 V3 arrays, following the manufacturer’s instructions and using default settings for hybridization, and performing background subtraction, normalization, and scatter plot analysis in GenomeStudio. Spontaneous in vitro differentiation into derivatives of the three germ layers was performed using conventional embryoid body differentiation as described [10]. Immunocytochemistry was carried out using standard procedures with paraformaldehyde fixation and using appropriate Alexa-conjugated secondary antibodies (Life Technologies). Primary antibodies used were anti-SMA (Dako #M0851, 1:100), anti-AFP (Dako #A0008, 1:300), and anti-βIII-tubulin (Sigma #T8660, 1:1000). One LQT3 and one WT hiPSC line showing near-complete transgene silencing and overall hESC-like characteristics according to these assays were used for further investigation.

### Maintenance of hiPSCs

hiPSCs were routinely cultured in 6-well plates on 1:75 diluted Matrigel™ HC (Corning #354263), in FTDA medium [10]. FTDA consisted of DMEM/F12, 1× PenStrep/l-glutamine, 1× defined lipids (Life Technologies #21331020, #10378016, and #11905031, respectively), 0.1 % human serum albumin (Biological Industries #05-720-1B), 1× ITS (BD #354350), 10 ng/ml FGF2 (PeproTech #100-18B), 0.2 ng/ml TGFβ1 (eBioscience #34-8348-82), 50 nM Dorsomorphin (Santa Cruz #sc-200689), and 5 ng/ml Activin A (eBioscience #34-8993-85). Cells were routinely passaged as single cells or, initially, as clumps of cells. For single cell splitting, cells were grown to full confluence (until cultures seemingly appeared syncytial), digested for 10–15 min using Accutase™ (Millipore #SCR005) with 10 µM Y27632 (abcamBiochemicals #ab120129), and replated in the presence of 10 µM Y27632 at 400,000–600,000 cells per well of a 6-well plate. hiPSCs reached confluence after 3 days under these conditions and were subsequently harvested as above, for continuous maintenance or for the induction of differentiation. hiPSCs were kept in culture for a maximum of 30 passages. Cell lines were tested negative for mycoplasma.

### Directed CM differentiation of hiPSCs

In some experiments, cardiomyocyte differentiation was induced using END-2 co-culture [17], by plating clusters of undifferentiated hiPSCs onto confluent END-2 feeders in Knockout™ DMEM (Life Technologies #10829018), 1× ITS (insulin/transferrin/selenium, BD #354350), 250 µM 2-phospho-l-ascorbic acid, and PenStrep/l-glutamine. For most experiments, hiPSCs were differentiated using a directed differentiation protocol [33]: fully confluent hiPSCs were digested with Accutase and 10 µM Y27632 for 10–15 min at 37 °C, and dissociated into single cells using a 1 ml pipette. Cells were pelleted and resuspended in d0 differentiation medium. d0 medium was composed of Knockout DMEM, 0.4 % polyvinyl alcohol (Sigma #363170), 10 µM Y27632, 1× ITS, 1× PenStrep/l-glutamine, 5 ng/ml FGF2, 0.5–2 ng/ml BMP4 (R&D #314-BP-050), and 1–2 µM CHIR99021 (AxonMedchem #Axon 1386). Cell concentration was adjusted to 40,000–80,000 cells per ml. 100 µl were added to each well of a 96 well V-bottom plate (Nunc #277143). EBs were allowed to form over night after a 1 min plate centrifugation step at 400 g. Next day (d1), EBs were washed in TS medium and transferred into ultra-low attachment 96 well U-bottom plates (Corning #7007). TS medium contained KO-DMEM, 1× TS, 1× lipid additive (Sigma #L5146), and 1× PenStrep/l-glutamine. 100× TS stock was prepared in advance by dissolving 55 mg transferrin (Sigma #T8158) in 100 ml PBS containing 0.067 mg sodium selenite (Sigma #S5261). On days 2–3, the EBs were incubated in TS medium together with 2 µM IWP-2 (Santa Cruz #sc-252928), followed by incubation in TS medium w/o IWP-2 hence after. Daily media changes were carried out under a stereo microscope using 200 µl pipettes with a wide opening. Spontaneous beating was commonly observed from day 6 onwards and scored in the 96-well plates. After the initial differentiation in multi-well plates, beating EBs were usually pooled and further maturated in 6-well plates with ultra-low attachment surface (Corning #3471). CM maintenance medium consisted of KO-DMEM, 2 % FCS, and 1× PenStrep/l-glutamine. Cardiomyocytes were typically analysed approximately 4 weeks after the initiation of differentiation.

FACS analysis of differentiated cultures was performed on Beckman Coulter Gallios instrumentation as described [33], following dissociation with 1× TrypLE Select (Life Technologies #12563011) and using PBS/0.5 % saponin/5 % FCS for all incubation steps (anti-CTNT, Labvision #MS-295-P, 1:150/Alexa-488-conjugated anti-mouse, Life Technologies #A11001). SCN5A expression in cardiomyocytes was monitored using RT-qPCR analysis (Table S2) or standard immunocytochemistry of dissociated hiPSC-CMs replated onto gelatin-coated dishes (anti-SCN5A, alomone labs #ASC-005, 1:150). Additional antibodies used were anti-α-actinin (Sigma #A7811, 1:800), and anti-NKX2.5 (R&D #AF2444, 1:100).

### Patch clamp analysis

For patch-clamp experiments beating aggregates after 4 weeks of differentiation were collected in PBS and dissociated with 1 mg/mL collagenase type B (Roche) for 60 min at 37 °C under shaking conditions. Isolated single cells were plated at low densities on fibronectin-coated (0.1 %) coverslips in differentiation medium. Patch-clamp recordings were performed after 48–72 h on single beating cardiomyocytes using an EPC10 amplifier (Heka) in the whole cell configuration.

Na^+^ current was measured in the voltage clamp mode. For recording of Na^+^ current peak and recovery from inactivation the internal solution contained (in mM) 3 NaCl, 133 CsCl_2_, 2 MgCl_2_, 2 NaATP, 2 TEACl, 10 EGTA and 5 Hepes, pH 7.3 (CsOH) and the external solution: 7 NaCl, 133 CsCl_2_, 1.8 CaCl_2_, 1.2 MgCl_2_, 5 Hepes, 11 glucose, 0.005 nifedipine, pH 7.4 (CsOH). Peak Na^+^ currents were measured in response to a −10 mV depolarizing pulse of 40 ms from a holding potential of −100 mV, normalised to the cell capacitance and expressed in pA/pF. For analysis of the recovery from inactivation kinetics, pairs of depolarization pulses from −100 to 10 mV were applied with increasing delays between the two pulses (from 1.5 to 57 ms) and the second peak Na^+^ current was normalized to the first, plotted against the delay and these values were fitted with a mono-exponential growth to obtain the time constant of recovery.

Action potential recordings were performed in the current clamp mode with an internal solution containing (in mM) 50 KCl, 80 K-Asparatate, 1 MgCl_2_, 3 MgATP, 10 EGTA, 10 Hepes, pH 7.4 (KOH) and an external solution containing 140 NaCl, 5.4 KCl, 1.8 CaCl_2_, 1 MgCl_2_, 10 Hepes, 10 glucose, pH 7.4 (NaOH). APs were elicited by 2.5 ms long current injection pulses though the patch pipette and the strength of the pulse was increased stepwise until stable action potential generation was established. The current injections were controlled by an external Stimulator (Model 2100, A-M Systems) attached to the EPC10 amplifier. To quantify the frequency-dependent AP duration, cardiomyocytes were stimulated at different pacing periods and at each pacing period the average action potential duration at 90 % of repolarization (APD_90_) was determined. APD_90_ values were plotted against pacing periods and a linear regression analysis was used to determine the slope of this relationship for each individual cell. The effects of mexiletine (100 µM), ranolazine (20 µM) and phenytoin (10 µM) on APD were recorded at a constant stimulation frequency of 0.6, 0.2, and 0.2 Hz, respectively. Data were acquired at a sampling rate of 10–20 kHz (voltage clamp) or 5 kHz (current clamp), digitized with the Patchmaster software (HEKA) and analysed offline using Fitmaster (HEKA) or Labchart software (AD Instruments). AP parameters were analysed with the cardiac action potential analysis module of Labchart. APD_90_ was calculated from the peak of the AP to the point where the AP had dropped by 90 % of its amplitude.

### Electrophysiological analysis on microelectrode arrays (MEAs)

Electrophysiological analysis on microelectrode arrays (USB-MEA256 system, Multichannel Systems) was performed essentially as previously described [32]. 9-well MEAs were coated with a small volume of 1:150 pre-diluted Matrigel/0.1 % gelatin solution in KO-DMEM for approximately 2 h at room temperature. hiPSC-CMs were dissociated from maintenance cultures using a 1× or 10× TrypLE Select digestion to obtain a single-cell suspension or small cell aggregates. Approximately 20,000 cells were plated onto the MEA surfaces in a ~3 µl droplet and allowed to attach for ~1 h. MEA chambers with attached cells were then filled with 200 µl of CM maintenance medium. Two days later, baseline recordings were performed at 37 °C. Only FP spectra showing a clear *T*_max_-like signal were considered. *T*_max_ and peak-to-peak finding algorithms were implemented in MC Rack software v4.5.7. Field potential durations (FPDs, QT_max_ intervals) and beating frequencies (RR intervals) were averaged from five consecutive measurements from independent recordings. Data were processed in MS Excel using Bazett’s formula for frequency correction: FPD (cQT_max_) = QT_max_ (ms)/(RR (s))^0.5^. Only samples showing beat intervals in the range of ~700–2300 ms were considered for QT_max_ quantification. Recordings of drug-treated cells were initiated after a wash-in time of about 10 min. Wash-out recordings were performed after three to five media changes. FP curves monitoring drug responses were overlaid using Adobe Photoshop. Mexiletine was administered at 5–20 µM, as indicated in figures. Ranolazine was used at 20 µM.

### Statistics

Electrophysiological data are presented as mean values from biological replicates ± SEM. ECG-based data are presented as mean values ± SD. Statistical analysis was performed using appropriate paired and unpaired 2-sided Student’s *t* test or Fisher’s exact test F. A *p* value of <0.05 was considered statistically significant. * In figures indicates *p* < 0.05, and ** denotes *p* < 0.01.

## Results

### Clinical history of a family with LQT3 syndrome

**Fig. 1:**
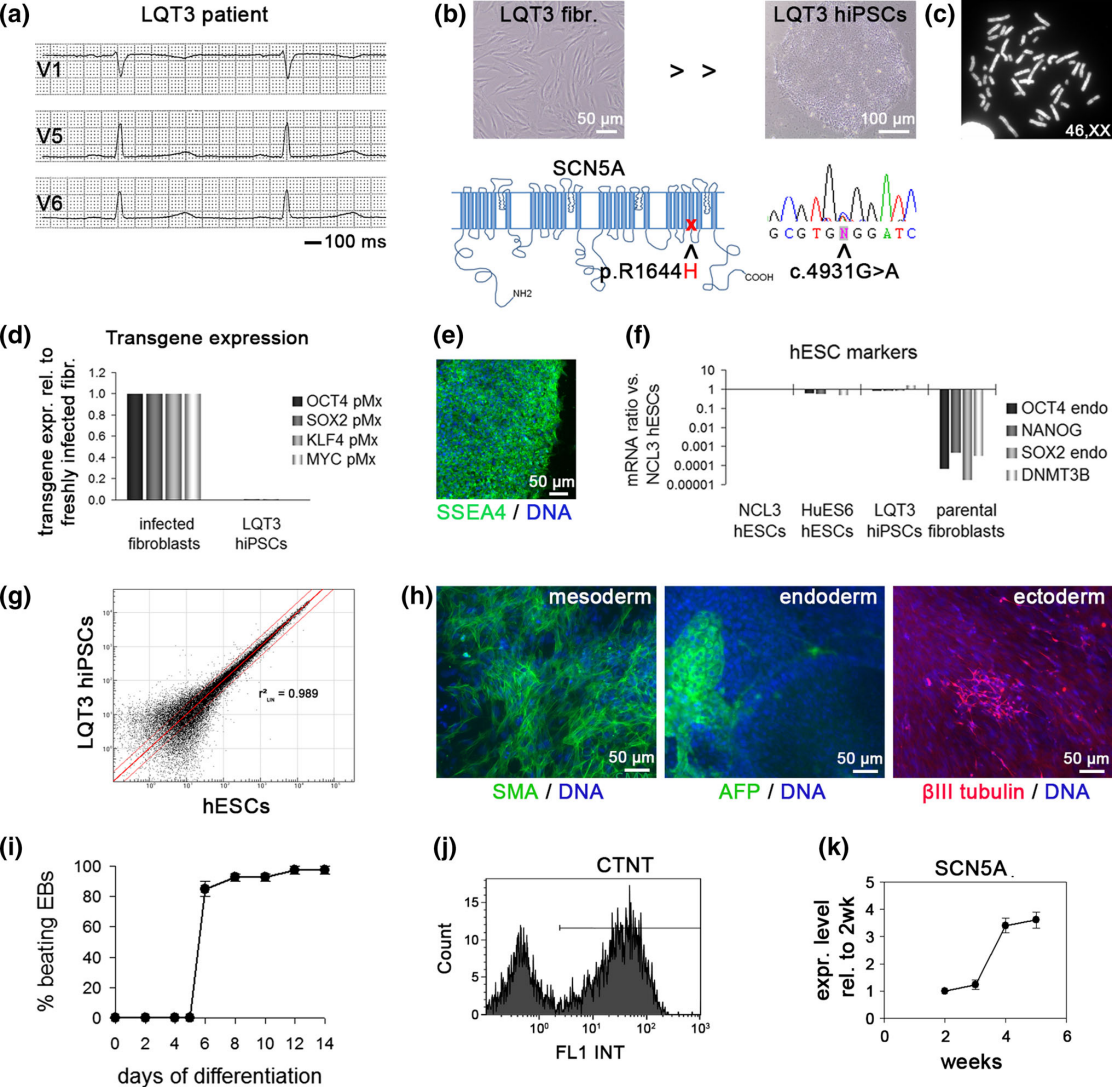
Generation and characterization of R1644H hiPSCs. **a** Electrocardiogram of the donor LQT3 patient displaying QT prolongation (QTc: ~507 ms). **b** Phase contrast morphology of LQT3 skin fibroblasts and reprogrammed hiPSCs (*top*). *Bottom* Illustration of amino acid substitution in SCN5A (*bottom left*) and sequencing confirmation of underlying heterozygous c.4931G>A nucleotide exchange at the DNA level (*bottom right*, reverse complement strand). **c** R1644H hiPSCs have a normal karyotype (*n* = 10). **d** RT-qPCR analysis of retroviral transgene expression in freshly infected LQT3 fibroblasts and LQT3 hiPSCs. **e** Immunofluorescence analysis of the pluripotency marker SSEA4 in LQT3 hiPSCs. **f** RT-qPCR expression analysis of endogenous pluripotency genes in LQT3 hiPSCs, in comparison to two hESC lines and the parental fibroblasts. **g** Scatter plot analysis of microarray gene expression data from LQT3 hiPSCs and NCL3 hESCs. Note the high global similarity indicated by linear regression analysis. *Red lines* denote intervals of twofold changes in gene expression. **h** Immunofluorescence analysis of spontaneous differentiation into derivatives of the three germ layers. *SMA* smooth muscle actin, *AFP* alpha-fetoprotein. **i** Percentage of beating EBs over time generated from LQT3 hiPSCs through directed differentiation (*n* = 2). **j** Representative FACS analysis of differentiated LQT3 EBs indicating the cardiomyocyte fraction based on cardiac Troponin T (CTNT) staining. **k** RT-qPCR time-course analysis of SCN5A expression during cardiac differentiation of LQT3 hiPSCs. Cells were differentiated on END-2 feeders. Data are normalized against pan-cardiac markers to account for differences in CM yield between samples (*n* = 3)

Following a case of sudden infant death in a large family with congenital LQTS, 15 of 23 available family members were identified as heterozygous carriers of a c.4931G>A missense mutation in the *SCN5A* gene, a previously described LQT3-causing defect. In the sodium channel protein, this mutation promotes an arginine-to-histidine exchange at the cytoplasmic face of the D4S4 transmembrane segment (p.R1644H; Fig. 1b) [2]. R1644H was one of the first LQT3-causing mutations identified and has been shown to impair fast Na^+^ channel inactivation, thereby giving rise to the a persistent late sodium inward current [12, 30, 31].

The mean baseline QTc interval of LQT3 mutation carriers in this family was 478 + 35 ms (*n* = 14), including two patients with normal QTc values (410 and 424 ms; Table S1). There were no clinical signs for conduction disease or Brugada syndrome in the affected family members. Apart from the sudden cardiac death victim (2 month-old, unknown genotype), six LQT3 patients had syncope before medical treatment (mean age of first event: 24 years). 10 of the patients were treated with β- blockers, and six were given class I antiarrhythmics (mexiletine or phenytoin—three of these with concomitant β-blocker medication). Cardiac devices were implanted in two LQT3 patients that refused oral therapy due to bradycardia and/or orthostatic intolerance (Table S1) [34].

### Generation and characterization of LQT3 hiPSCs

One affected family member presenting with QT interval prolongation was selected for hiPSC derivation (Fig. 1a; #5-3 in Table S1). Primary fibroblasts were derived from a skin biopsy taken from the patient following ethical approval and informed written consent. The presence of the mutation was confirmed by DNA sequencing in the cultured cells (Fig. 1b). hiPSCs were derived from these using standard retroviral reprogramming methodology [13, 28]. An hiPSC clone with normal karyotype (Fig. 1b, c) was selected for further characterization. Transgenes of the four reprogramming factors were silenced to negligible levels in LQT3 hiPSCs (Fig. 1d). LQT3 hiPSC colonies under stem cell maintenance conditions stained positive for the surface human embryonic stem cell (hESC) marker SSEA4 (Fig. 1e). RT-qPCR analysis suggested full activation of endogenous hESC marker gene expression (Fig. 1f). Global expression profiling revealed that the transcriptome of LQT3 hiPSCs was virtually indistinguishable from that of hESCs (Fig. 1g). Spontaneous in vitro differentiation gave rise to derivative cell types of the three germ layers suggesting acquired pluripotency (Fig. 1h). Using a directed differentiation protocol [33], LQT3 hiPSCs robustly converted into spontaneously contracting cardiomyocytes, as evidenced by high percentages of beating embryoid bodies (EBs) as well as FACS analysis for cardiac troponin C (Fig. 1i, j; Movies S1, S2). Finally, because early hiPSC-derived cardiomyocytes tend to be immature in their physiological properties [33], a time-course expression analysis of *SCN5A* was performed. This revealed that *SCN5A* was expressed at lower levels in early (~2 week-old) hiPSC-CMs, but at higher and stable levels after approximately 4 weeks of culture (Fig. 1k). These data suggested that LQT3 hiPSCs had acquired a fully reprogrammed hESC-like state and that hiPSC-CMs could be functionally analysed following several weeks of in vitro culture.

As control, wild-type (WT) hiPSCs were derived from an unrelated, healthy donor and characterized in a similar manner. Briefly, WT hiPSCs displayed a hESC-like morphology and karyotype under stem cell maintenance conditions (Fig. S1a, b), had fully silenced the exogenous transgenes (Fig. S1c), and expressed endogenous marker genes at hESC-like levels (Fig. S1d). WT hiPSCs differentiated into derivatives of the three germ layers upon spontaneous in vitro differentiation (Fig. S1e), and readily formed cardiomyocytes using independent differentiation protocols (Fig. S1f, g; Movies S3, S4). Hence, based on these assays, WT hiPSCs shared key pluripotency features with LQT3 hiPSCs and differentiated into cardiomyocytes at comparable efficiencies.

### Electrophysiological phenotypes of R1644H hiPSC-CMs

**Fig. 2:**
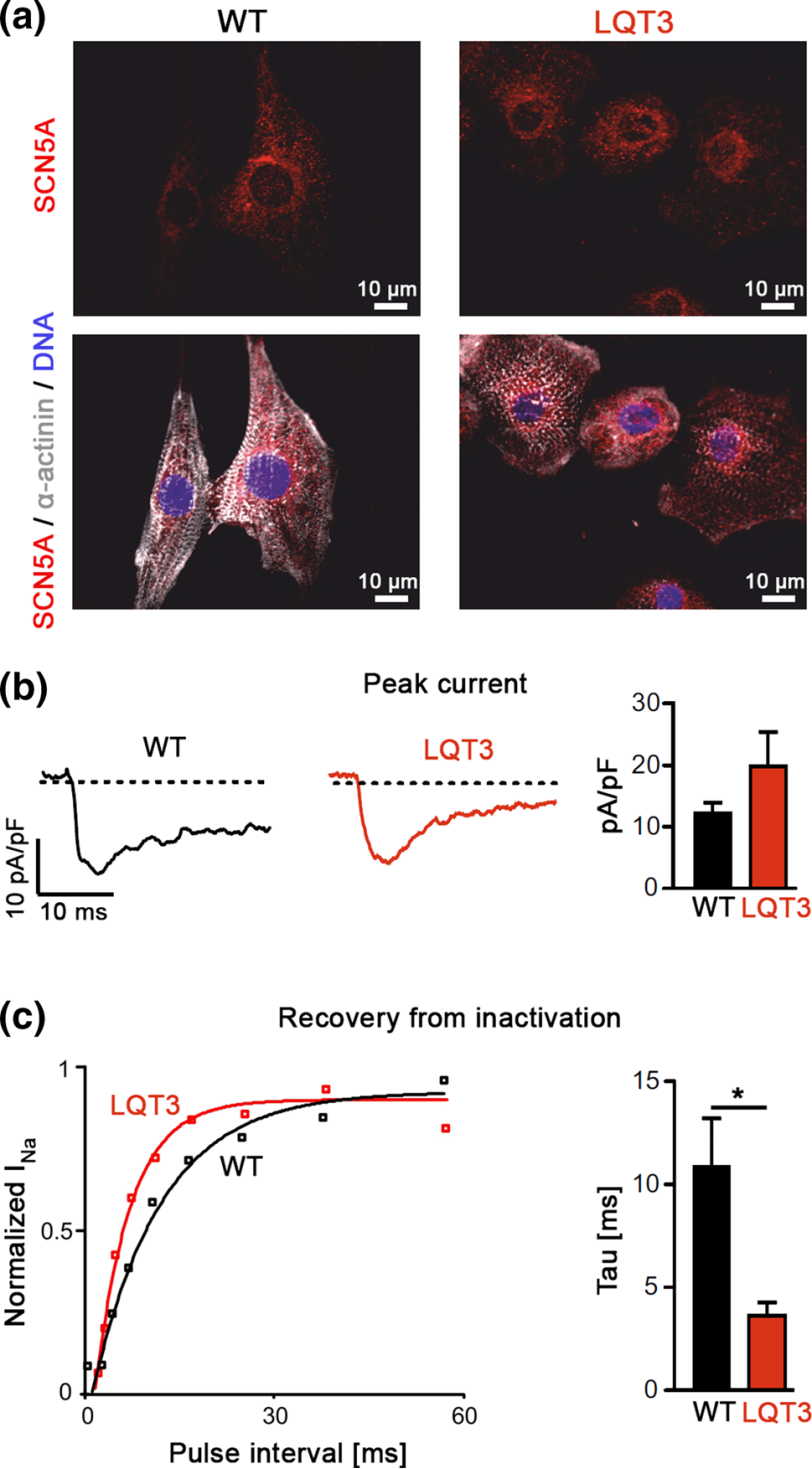
Sodium channel function in LQT3 and control hiPSC-CMs. **a** Immunofluorescence stainings of SCN5A (*red*) in CMs derived from LQT3 and WT hiPSCs show perinuclear and partial outer membrane localization. **b** Representative peak sodium current traces (*left*) and average peak current densities (*right*, *n* = 11 WT, *n* = 6 LQT3, n.s.). **c** Analysis of recovery from inactivation using a 2-pulse protocol. *Left* Examples of normalized sodium currents plotted against pulse intervals. Note the accelerated recovery in the LQT3 hiPSC-CM. *Right* Averaged time constant of recovery from inactivation (*n* = 13 WT and *n* = 5 LQT3, *p* < 0.05)

We next investigated the electrophysiological characteristics of LQT3 and WT hiPSC-CMs. The SCN5A protein could be detected by immunocytochemistry in a large fraction of cardiomyocytes from both cell lines (Fig. 2a). Peak Na^+^ current density measurements at −10 mV using patch clamp recordings confirmed that the channel was functionally active in both WT and LQT3 hiPSC-CMs. Peak current amplitudes tended to be slightly increased in LQT3 cardiomyocytes, but this difference was not statistically significant (Fig. 2b, WT: 12.2 ± 1.8 pA/pF, *n* = 11; LQT3: 19.9 ± 5.5 pA/pF, *n* = 6). A clear increased recovery from inactivation of the sodium current was found in in LQT3 compared to WT-CMs (Fig. 2c; time constant τ of recovery from inactivation: WT 10.8 ± 1.8 ms, *n* = 13; LQT3: 3.6 ± 0.6 ms, *n* = 5, *p* < 0.05). These data demonstrate that the pathognomonic electrophysiological feature of the R1644H mutation was found in our LQT3 disease model [30].

**Fig. 3:**
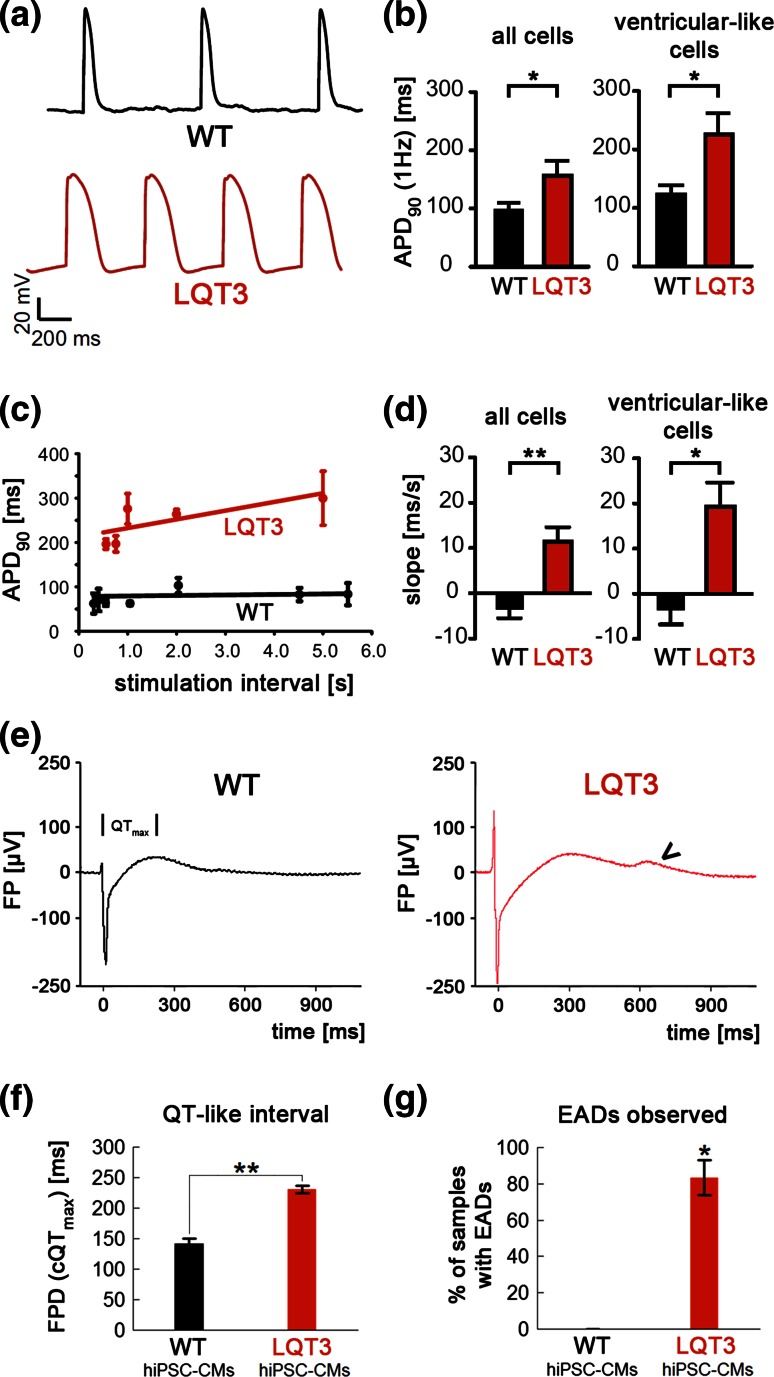
APD and FPD phenotypes of LQT3 hiPSC-CMs. **a** Representative APs from WT and LQT3 hiPSC-CMs. **b** APD_90_ quantification at a stimulation frequency of 1 Hz. LQT3 hiPSC-CMs showed a significant prolongation (all cells: *n* = 14 WT, *n* = 27 LQT3, *p* < 0.05; ventricular-like cells: *n* = 7 WT, *n* = 15 LQT3, *p* < 0.05). **c** Action potential restitution (APD_90_/pacing period relationship) in a representative WT (*black*) and LQT3 (*red*) hiPSC-CM. Note the positive slope in the LQT3 cell. **d** Statistical quantification of the slope of APD restitution in WT and LQT3 hiPSC-CMs (all cells: *n* = 8 WT, *n* = 17 LQT3, *p* < 0.01; ventricular-like cells: *n* = 5 WT, *n* = 8 LQT3, *p* < 0.05). **e** Representative field potential recordings of WT and LQT3 hiPSC-CM clusters using MEAs. FPD (QT_max_) was quantified on the basis of Q and maximum T wave-like signals (see indicated interval). The* arrowhead* marks a typical EAD-like signal observed in LQT3 CMs. **f** FPD quantification from independent WT and LQT3 hiPSC-CM preparations (*n* = 3 WT, *n* = 5 LQT3, *p* < 0.01). **g** Field potentials with EADs were observed in a high percentage of LQT3 samples (*n* = 7 WT, *n* = 17 LQT3, *p* < 0.05)

Next, we explored action potential durations (APD) in the current clamp mode. APDs measured at a stimulation frequency of 1 Hz were increased in LQT3 hiPSC-CMs (Fig. 3a). This was true for averaging data from all types of cells (Fig. 3b, left: APD_90_ WT: 95.1 ± 12.3 ms, *n* = 14; LQT3: 155.7 ± 25.7 ms, *n* = 27, *p* < 0.05), whereas the phenotype became somewhat more pronounced when confining the analysis to ventricular-like cells (Fig. 3b, right: APD_90_ WT: 122.9 ± 15.2 ms, *n* = 7; LQT3: 225.5 ± 36.6 ms, *n* = 15, *p* < 0.05). Moreover, as a hallmark of LQT3, APD is highly frequency dependent and expected to increase at low beating rates, as shown previously [8, 16]. Hence, APD was determined in individual hiPSC-CMs at various stimulation frequencies (see examples given in Fig. 3c). The APD restitution slope (dependency of APD on the pacing period) was determined through linear regression analysis. Analysis across all cardiac subtypes yielded a steep APD restitution slope for LQT3 hiPSC-CMs (+11.4 ± 3.2 ms/s, *n* = 17; Fig. 3d, left) indicating prolonged APD especially at low stimulation rates, whereas this dependency was absent in WT hiPSC-CMs (−3.2 ± 2.2 ms/s, *n* = 8). The difference became even more accentuated when including only cells with ventricular-like AP shapes into the analysis (Fig. 3d, right, WT: −3.2 ± 3.5 ms/s, *n* = 5; LQT3: +19.3 ± 5.3 ms/s, *n* = 8).

Further, we used bulk cultures of hiPSC-CMs on microelectrode arrays (MEAs) in order to explore, whether these pathognomonic features of LQT3 could also be detected using an independent assay based on extracellular field potential recordings. Clear T wave-like signals were readily detectable with both WT and LQT3 CMs, permitting field potential duration (FPD) measurements (Fig. 3e). Frequency-corrected FPDs determined under spontaneous beating conditions were significantly increased in LQT3 hiPSC-CMs compared to WT cells (Fig. 3f). Interestingly, most FP traces from LQT3 hiPSC-CMs displayed notched T wave-like signals, which we attribute to EADs evoked by reactivating Na^+^ currents in the mutant cells (arrow head in Fig. 3e). Quantification from independent cell preparations revealed that these EAD signals were highly reproducible in LQT3 hiPSC-CMs but never seen in WT cells (Fig. 3g). Collectively, these data reveal typical LQT3 features in R1644H hiPSC-CMs by electrophysiological analysis, including EADs at high probability.

### Pharmacological rescue of LQT3 phenotypes

**Fig. 4:**
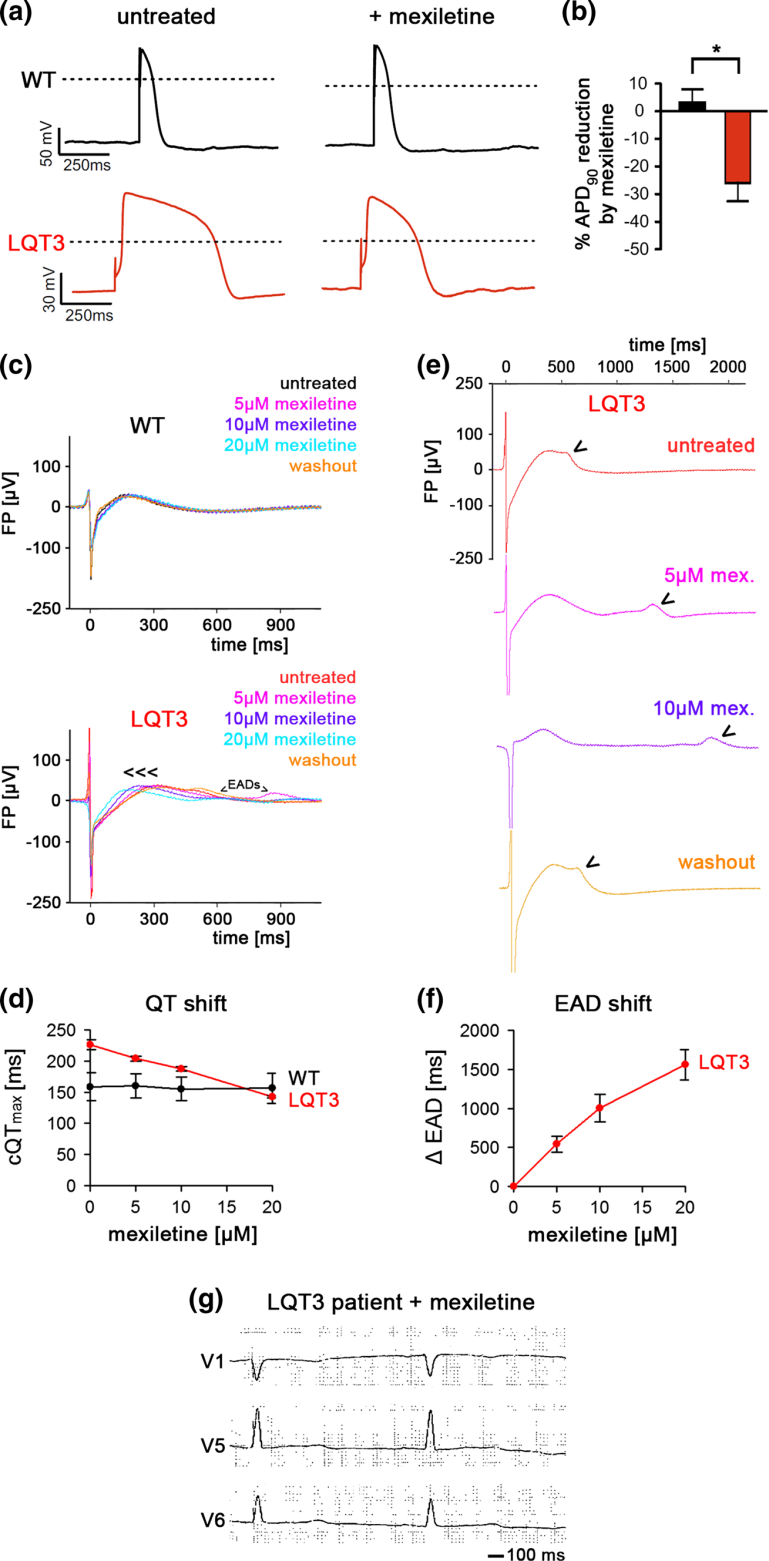
Rescue of disease-specific phenotypes in LQT3 hiPSC-CMs by mexiletine. **a** Representative action potential traces before (*left*) and after (*right*) mexiletine treatment (100 µM). Note the AP shortening following drug administration in the LQT3 cells. **b** Quantification of mexiletine-induced APD_90_ reduction (*n* = 8 WT, *n* = 5 LQT3, *p* < 0.05). **c** Representative field potential recordings showing that mexiletine reduces FPD specifically in LQT3 hiPSC-CMs but not in WT cells. **d** Quantification of mexiletine effect at different dosages on FPD in WT and LQT3 hiPSC-CMs (*n* = 3). **e** Mexiletine shifts EADs in LQT3 hiPSC-CMs towards later time-points in a dose-dependent and reversible manner (representative MEA traces). *Arrowheads* mark EADs. **f** Average quantification of induced EAD shift in FPs of LQT3 hiPSC-CMs as dependent on mexiletine dosage (*n* = 3). EADs were fully suppressed using >20 µM of mexiletine. **g** Electrocardiogram of the donor LQT3 patient under mexiletine monotherapy (2 × 100 mg/day; QTc: ~440 ms)

Likely, the above phenotypes are a direct consequence of the reactivating sodium current in R1644H CMs. To demonstrate this, and to assess the predictability of our model for drug screening, we employed mexiletine, a Na^+^ channel inhibitor commonly used in LQT3 therapy [20]. Using patch clamp analysis, we found that mexiletine (100 µM) significantly reduced APD in LQT3 hiPSC-CMs (−25.8 ± 6.7 %, *n* = 5, *p* < 0.05), thereby correcting the APD prolongation phenotype at the single cell level (Fig. 4a, b). Importantly, this effect was not seen in WT cells (APD: +3.2 ± 4.7 %, *n* = 8) suggesting that this drug preferentially acts on the reactivated sodium current (Fig. 4a, b). Similarly, mexiletine reduced the FPD in bulk cultures of LQT3 hiPSC-CMs, in a dose-dependent manner, and showed virtually no effect in WT cells (Fig. 4c). 10–20 µM of mexiletine were sufficient to reduce FPDs of LQT3 CMs to WT-like values (Fig. 4d). Interestingly, we noticed that the EADs were also affected by mexiletine treatment of LQT3 hiPSC-CMs. In a highly reproducible and dose-dependent manner, mexiletine administration progressively shifted the EAD signal towards later time points, outside the T wave-like repolarization window, and doses above 20 µM fully suppressed EADs (Fig. 4e, f). To substantiate the idea that late sodium current blockage was the basis for these patient-specific effects, we additionally evaluated alternative drugs, ranolazine and phenytoin, acting via the same principle [3, 25]. Indeed, both compounds specifically reduced APDs in LQT3 hiPSC-CMs (phenytoin: −29.7 ± 9.2 %, *n* = 6; ranolazine: −26.3 ± 9.2 %, *n* = 7) but not in WT cells (Fig. S2 a–d). Similarly, ranolazine administration (20 µM) abolished spontaneous EADs in patient hiPSC-CMs and caused significant LQT3-specific FPD shortening (Fig. S2e, f).

Interestingly, these data provide support of the therapeutic strategy applied to the affected LQT3 family members, including the individual who donated cells for hiPSC derivation (Table S1). Interpretation of these data is, however, complicated by the fact that several patients of this family were treated both with antiarrhythmics and beta blockers. As a tendency, though, beta blocker monotherapy was applied to family members with a baseline QTc below 500 ms and this caused a rather moderate decline (17 ± 4 ms, *n* = 3). In comparison, patients with a baseline QTc > 500 ms were treated with antiarrhythmics (mexiletine or phenytoin) ± additional beta blocker, which caused a more pronounced QTc reduction (62 ± 18 ms, *n* = 5, *p* < 0.01 vs. beta blocker monotherapy). In support of this notion, the patient underlying our study showed a QTc reduction from ~507 to ~440 ms following mexiletine monotherapy (2 × 100 mg/day, Fig. 4g), whereas additional treatment with bisoprolol only had a slight additive effect (~435 ms, Table S1). Using this strategy, adverse cardiac events were prevented in a sustained manner in all patients who had experienced syncope prior to therapy (Table S1).

## Discussion

Mutations in SCN5A can give rise to distinct disease phenotypes, namely LQT3, Brugada syndrome, progressive cardiac conduction disease, and sinus node diseases [18]. Moreover, LQT3 may be caused by diverse gain-of-function mutations in *SCN5A* and, as a result of this fact, not all patients respond to a given pharmacological treatment at similar efficacy [19]. Patient-specific hiPSC-CMs can be utilized for evaluating putative disease-correcting effects of drugs, as exemplified with models of LQT2 and JLNS [14, 32]. However, because electrophysiological analysis at the single-cell level is technically challenging and because hiPSC-CMs present a heterogeneous mixture of cardiac subtypes, more integrated and disease-associated readouts are desirable. EADs are considered triggers of life-threatening Torsade de Pointes (TdP) tachycardia in LQTS, as they may contribute to increased dispersion of ventricular repolarization [1]. Interestingly, on microelectrode arrays, prominent EADs were consistently observed in our LQT3 model but were never seen in WT controls, neither in the cell line used here nor in other independent hiPSC lines [32]. MEAs may be particularly reliable in detecting EADs as they tend to overcome cell-to-cell variation by averaging field potentials from many cells in a given preparation. Furthermore, intracellular ion concentrations in cardiomyocytes (Na^+^, K^+^, Ca^2+^) are not disturbed in MEA recordings, in contrast to the patch-clamp technique that dialyzes Na^+^, K^+^ against the electrode solution and usually buffers Ca^2+^. Perhaps for these reasons, we and others did not observe EADs in LQT3 hiPSC-CMs using patch-clamp analysis of single cells [15, 29]. It will be interesting, therefore, to see whether EADs are a universal feature also of CMs from other LQT3 hiPSC models, as revealed here using field potential recordings.

Mexiletine is a commonly used drug for treating LQT3 patients because it is more effective in inhibiting late Na^+^ currents than peak current density and therefore acts preferentially on the pathogenic feature of mutant channels [7, 26, 29]. This drug thereby appeared well-suited for balancing consequences of the R1644H mutation which causes a sustained, non-inactivating sodium current as a result of disperse reopenings following initial fast channel inactivation [7, 30]. Indeed, mexiletine specifically and dose-dependently reduced APDs and FPDs in R1644H hiPSC-CMs but had no effect on WT cells. Moreover, EAD-like signals arising specifically in field potential recordings from LQT3 hiPSC-CMs were shifted towards later time-points by mexiletine administration in a dose-dependent manner or were fully suppressed at higher concentrations. Thus, at least in the cellular model, even low dosages of mexiletine exerted beneficial effects by shifting EADs outside the critical repolarization time-window. Notably, the phenotype-correcting effects in LQT3 hiPSC-CMs were in accordance with the beneficial response to mexiletine administration in the LQT3 patient underlying our study. Moreover, alternative drugs counteracting the late sodium current, phenytoin and ranolazine, also reduced APDs/FPDs in our model. Indeed, phenytoin was successfully used to treat several affected members of our LQT3 family, as an alternative to mexiletine (Table S1). Hence, our data show that drug testing results from LQT3 hiPSC-CMs may be predictive for medical treatment.

This concordance between hiPSC and patient-based data is noteworthy because mexiletine treatment is not effective in all LQT3 patients and models [19]. For instance, Ma et al. analysed an independent hiPSC model of LQT3 and obtained only a partial rescue of the APD prolongation phenotype following high-dosage (50 µM) mexiletine treatment [15]. Moreover, Terrenoire et al. observed inhibitory side-effects on the hERG channel using mexiletine administration in yet a different LQT3 hiPSC model, which complicated medical interpretation [29]. We did not observe any effects on APD or FPD when monitoring mexiletine treatment of WT hiPSC-CMs, suggesting no side-effects at the moderate drug concentrations used in our study (≤20 µM). In summary, monitoring effects on FPD and EADs provides integrated readouts that might be most robust and predictive for investigating candidate drug responses in hiPSC models of LQT3.

## Electronic supplementary material

Fig. S1 Generation and characterization of hiPSCs from WT fibroblasts. (a) Undifferentiated phase contrast (left and middle panels) and stereo microscopic (right) morphology of biopsy-derived skin fibroblasts and reprogrammed WT hiPSCs. (b) WT hiPSCs have a normal karyotype (n = 10). (c) RT-qPCR analysis of retroviral transgene expression in freshly infected parental fibroblasts and WT hiPSCs. (d) RT-qPCR expression analysis of endogenous pluripotency genes in WT hiPSCs as compared to HuES6 hESCs (n = 3). (e) Immunofluorescence analysis of derivatives of the three germ-layers following spontaneous in vitro differentiation. AFP: alpha fetoprotein, SMA: smooth muscle actin. (f) Immunofluorescence staining of WT cardiomyocytes following END-2 co-culture differentiation. (g) Representative scoring of beating frequencies of WT EBs subjected to directed cardiac differentiation (TIFF 6618 kb)

Fig. S2 Patient-specific APD and FPD reduction by phenytoin and ranolazine. (a) Representative action potential traces before (left) and after (right) phenytoin treatment (20 µM). Note the AP shortening following drug administration in the LQT3 cells. (b) Quantification of phenytoin-induced APD_90_ reduction (n = 11 WT, n = 6 LQT3, p < 0.05). (c) Representative action potential traces before (left) and after (right) ranolazine treatment (10 µM). Note the AP shortening following drug administration in the LQT3 cells. (d) Quantification of ranolazine-induced APD_90_ reduction (n = 5 WT, n = 7 LQT3, p < 0.05). (e) Representative field potential recordings showing that ranolazine (20 µM) abolishes EADs and reduces FPD specifically in LQT3 hiPSC-CMs but not in WT cells (representative traces). (f) Quantification of ranolazine-induced FPD reduction in WT and LQT3 hiPSC-CMs (n = 3, p < 0.05 for LQT3) (TIFF 908 kb)

Movie S1 Spontaneously beating EB generated through directed differentiation of R1644H hiPSCs (AVI 5226 kb)

Movie S2 Spontaneously beating EB generated through differentiation of R1644H hiPSCs on END-2 feeders (AVI 12417 kb)

Movie S3 Spontaneously beating cluster generated through directed differentiation of WT hiPSCs (AVI 4825 kb)

Movie S4 Spontaneously beating cluster generated through differentiation of WT hiPSCs on END-2 feeders (AVI 7113 kb)

Table S1 Overview of available clinical data of LQT3 mutation carriers in the family underlying this study. The drug dosages are indicating daily medication. The hiPSCs used in this study were we derived from patient #5-3. BB: β-blocker. AA: antiarrhythmic drug. N/A: not available (PDF 204 kb)

Table S2 Primers used for conventional and RT-qPCR (XLSX 43 kb)
