# Supplementary figures and images for: Human iPS cell model of type 3 long QT syndrome recapitulates drug-based phenotype correction
Source: Basic Res Cardiol. 2016 Jan 23;111:14. doi: 10.1007/s00395-016-0530-0 (PMC4724360; doi:10.1007/s00395-016-0530-0)

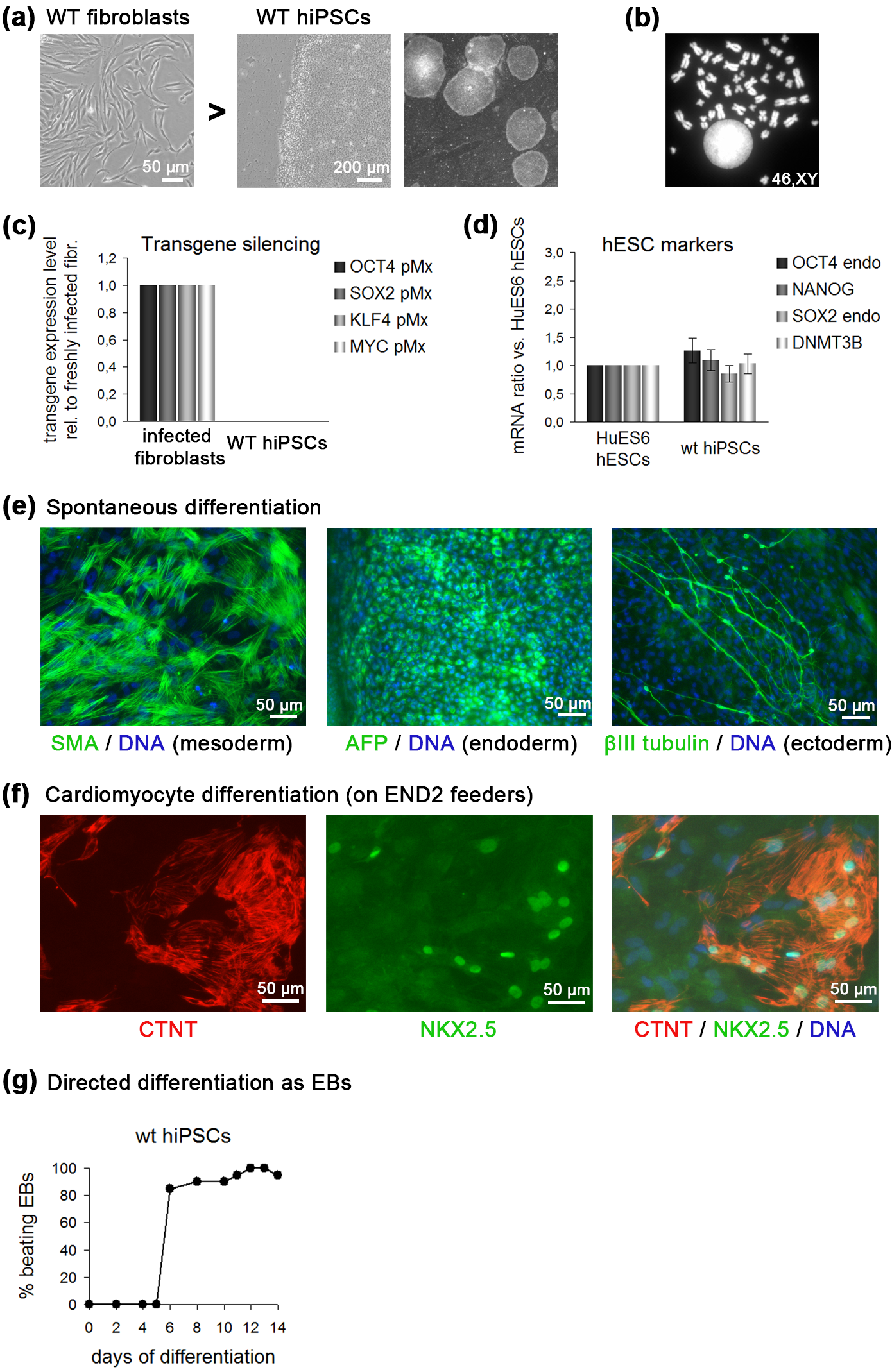

Supplement: Supplementary file 1 — Fig. S1 Generation and characterization of hiPSCs from WT fibroblasts. (a) Undifferentiated phase contrast (left and middle panels) and stereo microscopic (right) morphology of biopsy-derived skin fibroblasts and reprogrammed WT hiPSCs. (b) WT hiPSCs have a normal karyotype (n = 10). (c) RT-qPCR analysis of retroviral transgene expression in freshly infected parental fibroblasts and WT hiPSCs. (d) RT-qPCR expression analysis of endogenous pluripotency genes in WT hiPSCs as compared to HuES6 hESCs (n = 3). (e) Immunofluorescence analysis of derivatives of the three germ-layers following spontaneous in vitro differentiation. AFP: alpha fetoprotein, SMA: smooth muscle actin. (f) Immunofluorescence staining of WT cardiomyocytes following END-2 co-culture differentiation. (g) Representative scoring of beating frequencies of WT EBs subjected to directed cardiac differentiation (TIFF 6618 kb) [file 395_2016_530_MOESM1_ESM.tif]

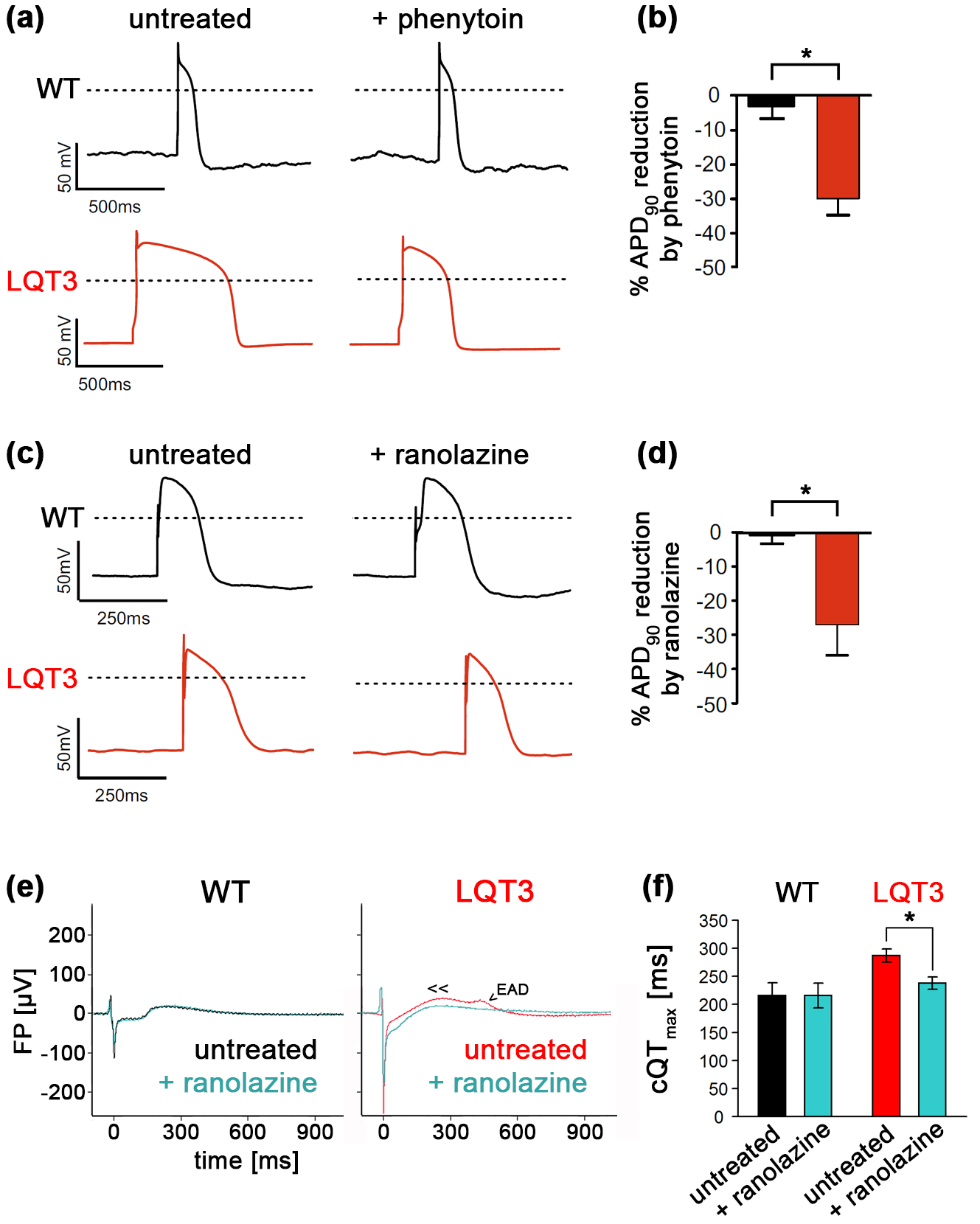

Supplement: Supplementary file 2 — Fig. S2 Patient-specific APD and FPD reduction by phenytoin and ranolazine. (a) Representative action potential traces before (left) and after (right) phenytoin treatment (20 µM). Note the AP shortening following drug administration in the LQT3 cells. (b) Quantification of phenytoin-induced APD90 reduction (n = 11 WT, n = 6 LQT3, p < 0.05). (c) Representative action potential traces before (left) and after (right) ranolazine treatment (10 µM). Note the AP shortening following drug administration in the LQT3 cells. (d) Quantification of ranolazine-induced APD90 reduction (n = 5 WT, n = 7 LQT3, p < 0.05). (e) Representative field potential recordings showing that ranolazine (20 µM) abolishes EADs and reduces FPD specifically in LQT3 hiPSC-CMs but not in WT cells (representative traces). (f) Quantification of ranolazine-induced FPD reduction in WT and LQT3 hiPSC-CMs (n = 3, p < 0.05 for LQT3) (TIFF 908 kb) [file 395_2016_530_MOESM2_ESM.tif]
