# Supplementary material for: Human iPS cell model of type 3 long QT syndrome recapitulates drug-based phenotype correction
Source: Basic Res Cardiol. 2016 Jan 23;111:14. doi: 10.1007/s00395-016-0530-0 (PMC4724360; doi:10.1007/s00395-016-0530-0)
Supplement: Supplementary file 7 — Table S1 Overview of available clinical data of LQT3 mutation carriers in the family underlying this study. The drug dosages are indicating daily medication. The hiPSCs used in this study were we derived from patient #5-3. BB: β-blocker. AA: antiarrhythmic drug. N/A: not available (PDF 204 kb) [file 395_2016_530_MOESM7_ESM.pdf]

ID	Age Gender	Symptoms Age at first occurrence	Therapy and device implantation BB (β-blocker) AA (Class I antiarrhythmic drugs)	Basal QTc (ms)	QTc during therapy (ms)	ΔQTc (ms)
5-1	58 female	Syncope 29J	1986-2006: BB (metoprolol, 2x50 mg)<br>1994: pacemaker implantation due to bradycardia and orthostatic intolerance<br>Since 2006: AA (phenytoin, 1x50 mg alternatively mexiletine, 2x50 mg)	531	485 (AA)	**46**
5-3	23 female		Before 2006: BB (bisoprolol)<br>2006-2011: AA (mexiletine, 2x100 mg)<br>Since 2011: BB (bisoprolol, 2x 1.25 mg) + AA (phenytoin, 2x50 mg)	507	440 (AA)	**67**
5-7	63 female	Syncope 12J	1990-1998: BB (metoprolol 3x 50 mg)<br>Since 1998: AA (mexiletine, 3x 50 mg)	502	460 (AA+BB)	**42**
5-9	38 male		None	424		
5-10	42 female	Syncope 27J	1990-1993: BB (metoprolol)<br>2003-2006: BB (propranolol)<br>Since 2006 AA (mexiletine, 2x 50 mg)	531	461 (AA)	**70**
5-11	35 female		Since 2004: BB (propanolol)<br>2004: AA (mexiletine, 2x 50 mg)	N/A	426 (AA+BB)	
5-12	25 female		N/A	460		
5-20	59 male		BB (metoprolol, 2x50 mg)	480	460 (BB)	**20**
5-22	42 female	Syncope 33J	2005-2006: BB, orthostatic intolerance<br>AA (mexiletine): not tolerated<br>2010: defibrillator implantation	489	476 (BB)	**13**
5-23	57 female		Since 2001: BB	462	N/A	
5-26	30 female	Syncope 15J	Since 2000: BB (metoprolol 3x50 mg)	479	460 (BB)	**19**
5-27	56 female	Syncope 27J	Before 2006: BB<br>Since 2006: AA (phenytoin, 2x50 mg)	504	418 (AA)	**86**
5-47	10 female		N/A	450	N/A	
5-49	12 male		N/A	469	N/A	
5-50	11 female		N/A	410	N/A	

Table S1 Overview of available clinical data of LQT3 mutation carriers in the family underlying this study. The drug dosages are indicating daily medication. The hiPSCs used in this study were we derived from patient #5-3. BB: β-blocker. AA: antiarrhythmic drug. N/A: not available.
